# Current gene therapy using viral vectors for chronic pain

**DOI:** 10.1186/s12990-015-0018-1

**Published:** 2015-05-13

**Authors:** Jean-Marc G Guedon, Shaogen Wu, Xuexing Zheng, Caroline C Churchill, Joseph C Glorioso, Ching-Hang Liu, Shue Liu, Lucy Vulchanova, Alex Bekker, Yuan-Xiang Tao, Paul R Kinchington, William F Goins, Carolyn A Fairbanks, Shuanglin Hao

**Affiliations:** Graduate Program in Molecular Virology and Microbiology, University of Pittsburgh, School of Medicine, Pittsburgh, PA 15213 USA; Department of Anesthesiology, New Jersey Medical School, Rutgers, State University of New Jersey, 185 S. Orange Ave., MSB, F-548, Newark, NJ 07103 USA; Department of Anesthesiology, University of Miami Miller School of Medicine, Miami, FL 33136 USA; Department of Pharmaceutics, University of Minnesota, Minneapolis, MN USA; Department of Ophthalmology, University of Pittsburgh School of Medicine, Room 1020 EEI, 203 Lothrop Street, Pittsburgh, PA 15213 USA; Microbiology and Molecular Genetics, University of Pittsburgh School of Medicine, 424 Bridgeside Point II, 450 Technology Drive, Pittsburgh, PA 15219 USA; Department of Neuroscience, University of Minnesota, Minneapolis, MN USA; Department of Cell Biology & Molecular Medicine, New Jersey Medical School, Rutgers, State University of New Jersey, Newark, NJ 07103 USA; Department of Neurology & Neuroscience, New Jersey Medical School, Rutgers, State University of New Jersey, Newark, NJ 07103 USA; Department of Physiology & Pharmacology, New Jersey Medical School, Rutgers, State University of New Jersey, Newark, NJ 07103 USA; Department of Pharmacology, University of Minnesota, 9-177 Weaver Densford Hall, 308 Harvard Street, Minneapolis, MN 55455 USA

**Keywords:** Pain, Viral vectors, Gene therapy

## Abstract

The complexity of chronic pain and the challenges of pharmacotherapy highlight the importance of development of new approaches to pain management. Gene therapy approaches may be complementary to pharmacotherapy for several advantages. Gene therapy strategies may target specific chronic pain mechanisms in a tissue-specific manner. The present collection of articles features distinct gene therapy approaches targeting specific mechanisms identified as important in the specific pain conditions. Dr. Fairbanks group describes commonly used gene therapeutics (herpes simplex viral vector (HSV) and adeno-associated viral vector (AAV)), and addresses biodistribution and potential neurotoxicity in pre-clinical models of vector delivery. Dr. Tao group addresses that downregulation of a voltage-gated potassium channel (Kv1.2) contributes to the maintenance of neuropathic pain. Alleviation of chronic pain through restoring Kv1.2 expression in sensory neurons is presented in this review. Drs Goins and Kinchington group describes a strategy to use the replication defective HSV vector to deliver two different gene products (enkephalin and TNF soluble receptor) for the treatment of post-herpetic neuralgia. Dr. Hao group addresses the observation that the pro-inflammatory cytokines are an important shared mechanism underlying both neuropathic pain and the development of opioid analgesic tolerance and withdrawal. The use of gene therapy strategies to enhance expression of the anti-pro-inflammatory cytokines is summarized. Development of multiple gene therapy strategies may have the benefit of targeting specific pathologies associated with distinct chronic pain conditions (by Guest Editors, Drs. C. Fairbanks and S. Hao).

## Part 1. Introduction

(Carolyn A. Fairbanks, Lucy Vulchanova, and Caroline C. Churchill, Corresponding author, Carolyn A. Fairbanks, carfair@umn.edu)

Chronic pain is a widely experienced broad spectrum of debilitating conditions that represent a significant global public health concern. The problem of chronic pain and the challenges of pain management have been comprehensively addressed by the Institute of Medicine report on Chronic Pain in the United States [1]. It is acknowledged that the currently available options for the treatment and management of chronic pain are limited due to significant adverse side effects, particularly of our most effective and most commonly used analgesics; NSAIDS and opioids [1]. It is noted that as the knowledge of mechanisms underlying the diversity of chronic pain conditions continues to expand, opportunities for very selective treatments will continue to emerge. Included in the category of specialized therapeutics to target specific pain conditions would be any of a number of gene therapy strategies that have been under development for at least twenty years and continue to emerge as knowledge is gained on specific pain mechanisms. Gene therapy approaches may be complementary to pharmacotherapy for several advantages. First, gene expression may be targeted to specific central nervous system (CNS) or peripheral nervous system (PNS) regions, dermatomes, cell populations, or even nuclei. Second, gene therapeutics may provide sustained long-term analgesia without need for repeated dosing (again dependent on the gene expression product). A common strategy to modify gene expression in the CNS and PNS has been the use of viral vectors. Several major categories of viral vectors have been utilized for different advantages with respect to addressing specific CNS or PNS dysfunction. The most commonly used viral vectors in studies of treatment of pain are based on recombinant adenovirus (AD), adeno-associated virus (AAV), lentiviral (LV) vectors, and herpes simplex virus (HSV)-based vectors [2,3], especially AAV and HSV. Two major categories that are in development for chronic pain treatments are featured by the authors in this combined review article.

### HSV-1

A non-replicating viral vector derived from the HSV-1 offers the advantage of large packaging capacity as well as natural mechanism of targeting of sensory neurons [4] resulting in episomal gene expression in the corresponding dorsal root ganglia and discrete dermatomal effects of expression of the gene product. This mechanism is of particular interest for potentially treating specific pain conditions. The HSV-1 viral vector strategy carrying the gene for pro-enkephalin has been widely shown pre-clinically to reverse manifestations of chronic pain in models of inflammation [5], pancreatitis [6], spinal nerve [7] and infraorbital [8] nerve ligation, and bone cancer [9]. A recent phase I clinical trial [10] has demonstrated safety and indicated effectiveness of increased expression of the pre-proenkephalin gene to treat focal intractable cancer pain. The use of the HSV-1 viral vector strategy has also been applied pre-clinically for evaluation of the effectiveness of other potential gene therapeutics including, but not limited to overexpression of GABA [11], anti-inflammatory cytokines [12,13] and antisense to NaV1.7 sodium channels [14]. It is anticipated that development of multiple gene-expression strategies using the HSV-1 approach may greatly benefit long-term management specifically of focal chronic pain [15].

### AAV

The AAV vectors have comparatively lower packaging capacity which is somewhat mitigated through the use of self-complementary genome approaches. However, AAVs have the advantage of very limited immune response, long-term gene expression, and broad tropism for a variety of cell types [4]. The AAV vectors have multiple serotypes with distinct tropisms for distinct cell types. Through selection of the serotype used and/or tissue-specific promoters it may be possible to selectively deliver therapeutic gene expression. Several AAV serotypes have been particularly useful for gene transfer to a broad spectrum of brain regions to correct loss-of-function enzyme deficiency disorders [16]. The use of different AAV serotypes for CNS gene therapy is also of interest to those developing novel treatments for Alzheimer’s disease [17], Parkinson’s disease [18], Huntington’s chorea [19], epilepsy [20] ALS [21] and chronic pain [22] among others. However, in contrast to the enzymatic deficiency disorders, these neurological disorders would likely benefit from more site-specific targeting of specific regions or brain nuclei [20] to optimize their effects.

As with the HSV-1 vector there have been a number of pre-clinical studies in chronic pain models using the AAV approach. For example, intraspinal delivery of rAAV2 carrying the gene for BDNF resulted in reversal of neuropathic pain behaviors induced by chronic constriction injury [23]. Intraganglionic injection of an AAV5 serotype carrying a gene for shRNA targeting NaV1.3 channels resulted in reduction in neuropathic pain behaviors evoked by spared nerve injury [24]. Intrathecal delivery in catheterized rats of self-complementary AAV8 serotype carrying the gene for the anti-inflammatory cytokine IL-10 resulted in reduction of neuropathic pain behaviors invoked by L5 spinal nerve ligation [25].

### Therapeutic development considerations

Some barriers to clinical translation of AAV-mediated gene therapy have been previously identified and discussed [26]. These include the presence of circulating anti-AAV antibodies that may preclude the effectiveness of systemic AAV therapy [27] as well as an apparent need for high systemic titers of AAV vectors in order to achieve therapeutic outcomes. Direct delivery to the CNS through a spinal or intracerebroventricular route presents an opportunity to partially overcome these barriers as it has yielded higher levels of expression with lower viral titers compared to vascular routes of administration and it takes advantage of the relatively immuno-privileged status of the CNS, thereby reducing the risk of circulating anti-AAV neutralizing antibodies [27]. Translational development of direct CNS delivery of AAV vectors for therapeutic management of chronic pain requires consideration of the biodistribution of viral particles, which is partially determined by cellular tropism. It has been observed that AAV serotypes differ in their ability to transduce sensory neurons and in some cases target preferentially subsets of sensory neurons [28,29]. Importantly, there is also evidence for species differences in the cellular tropism of AAV serotypes [30]. Additionally, supra-spinal gene expression has been observed following intrathecal delivery of AAV5 [31], AAV6 [29] and AAV9 [32] serotypes.

#### Biodistribution

The principle that pharmacotherapeutics originally developed for systemic administration must be re-evaluated specifically for spinal delivery, has been long established [33] . There are unique features to the spinal route of administration that require independent safety assessment. Compared to pharmacotherapeutics and other constituents of CNS formulations, very little is known regarding the pharmacokinetics of spinally delivered AAV vectors. A number of factors that can greatly influence distribution of spinally delivered pharmacotherapeutics include, but are not limited to, the formulation constituents, the baricity of the formulation, and the positioning and/or state of consciousness of the subject [34]. It is likely to be the same for viral particles, though the spinal distribution has been less comprehensively evaluated compared to small molecules. A recent study [30] of the biodistribution of AAV8 vector genomes following intrathecal delivery in dog confirms a rostral caudal distribution at all levels of dorsal root ganglia and spinal cord, some detection in brain and evidence of systemic redistribution by detection of vector genomes in liver and spleen. The impact of distribution to the systemic circulation following intrathecal delivery, and in particular gene transfer to the liver [27,29-32] is not yet known.

#### Neuropathology

Having articulated the potential benefits of using a central route of administration to delivery of gene therapeutics, it is equally important to emphasize the need for assessment of potential neurotoxicity in pre-clinical models [33,35]. It has been previously observed for both AAV9 [36] and AAV8 [30] that carrying a gene that is foreign to the host (e.g. Green Fluorescent Protein) can elicit a significant pathological neuroimmune response that may, in fact, result in immunoneutralization or reduced free concentrations of the gene product, such as IL10 [30]. Given the expected advantage of long-term expression of gene therapeutics, evaluation in well-validated models of spinal neurotoxity of tissue exposed to the viral particles and consequent gene expression must be performed with consideration to the function of the specific cell types transduced (e.g. neurons, or glia, or choroid plexus cells, and/or meningeal cells).

In summary, the complexity of chronic pain states and the challenges of current pain management strategies call for development of unique approaches complementary to conventional pharmacotherapy. Development of multiple gene therapy strategies may have the benefit of targeting specific pathologies associated with distinct chronic pain conditions. There is much opportunity for discovery in this developing area; the present collection of articles features three distinct gene therapy approaches targeting specific mechanisms identified as important in the specific pain conditions modeled.

## Part 2. AAV mediated transfer of Kv1.2 sense RNA to primary sensory neurons: a potential strategy to treat peripheral neuropathic pain

(Shaogen Wu, Alex Bekker, Yuan-Xiang Tao, Corresponding author: Dr. Yuan-Xiang Tao, yt211@njms.rutgers.edu)

*Neuropathic pain, a common clinical problem, is often refractory to treatment with available therapies in part due to the incomplete understanding of the mechanisms that underlie induction and maintenance of this disorder. Nerve injury-induced abnormal spontaneous activity in the neuroma and primary sensory neurons is thought to contribute to neuropathic pain genesis. Voltage-gated potassium (Kv) channels, which are critical for establishing resting membrane potential and controlling neuronal excitability, are potential targets for treating neuropathic pain. This review focuses on current evidence on the role of Kv1.2, one of the α subunits in the Kv channel family, in neuropathic pain. We describe nerve injury-induced downregulation of Kv1.2 at the transcriptional and translational levels in primary sensory neurons of dorsal root ganglion. We also discuss how peripheral noxious stimulation induces such downregulation under neuropathic pain conditions. We finally present the evidence that rescuing Kv1.2 downregulation through adeno-associated virus mediated transfer of Kv1.2 sense RNA into the dorsal root ganglion may be a potential application in prevention and treatment for neuropathic pain.*

### Keywords

*Potassium channels, Kv1.2, dorsal root ganglion, adeno-associated virus, gene transfer, neuropathic pain.*

### P2.1.Introduction

Neuropathic pain is a public health problem that affects approximately 7-10% of the general population in the USA and Europe [37-39]. It is a cause of grave physiological and psychological distress in those affected, and it places significant pressures on the health care system. Billions of US dollars are spent on neuropathic pain related health care expenses, and many patients experience a loss of productivity. Neuropathic pain is caused by tissue damage or a disease that affects the somatosensory system [40,41]. It is characterized by ongoing or intermittent burning pain, an exaggerated response to noxious stimuli (hyperalgesia), and pain in response to normally innocuous stimuli (allodynia) [40,41]. Despite recent advancements in our fundamental understanding of neuropathic pain, there are still very few treatment options. Most of the conventional painkillers either do not relieve neuropathic pain or have serious side effects. Current approaches to tackle this disorder (e.g., opioids and other strong painkillers) temporarily reduce the sensation of pain, but do not treat the underlying pathology. Thus, uncovering the mechanisms of neuropathic pain may provide new strategies for the prevention and/or treatment of this disorder.

One of the common primary causes of peripheral neuropathic pain is abnormal spontaneous activity that arises in neuromas at the sites of nerve injury and also in dorsal root ganglion (DRG) neuronal bodies [40,41]. Abnormal excitability of the injured DRG neurons may result from changes in the expression and functional characteristics of receptors, enzymes, and voltage-dependent ion channels in the DRG [42-44]. Voltage-gated K^+^ (Kv) channels, which are critical for establishing resting membrane potential and controlling excitability of DRG neurons, are key players during these changes [45-47]. Application of Kv antagonists to sensory axons and to sites of ectopic afferent discharge facilitates ectopic firing [48-51]. Injection of these antagonists into nerve-end neuromas provokes intense pain [52]. Furthermore, knockdown of DRG Kv channels leads to abnormal mechanical pain [47,53]. Dramatic reductions in K^+^ currents and the expression of Kv channel mRNA and protein (e.g., Kv1.2) are observed in the injured DRG following peripheral nerve injury [45,46,54-59]. These findings indicate that a nerve injury-induced reduction in DRG Kv channels may contribute to neuropathic pain genesis.

In this article, we focus on current evidence on the role of Kv1.2, one of the α subunits in the Kv channel family, in neuropathic pain. We first review the nerve injury-induced downregulation of Kv1.2 at the transcriptional and translational levels in DRG. We then discuss how peripheral noxious stimulation induces such downregulation under neuropathic pain conditions. We finally present the evidence that rescuing DRG Kv1.2 downregulation through adeno-associated virus (AAV) mediated transfer of Kv1.2 sense RNA may be a potential application in prevention and treatment of neuropathic pain.

### P2.2. Expression and distribution of Kv1.2 in DRG after peripheral nerve injury

The Kv1.2 subunit participates in the formation of Kv channel tetramers in most DRG neurons. The mRNA for Kv1.1 and Kv1.2 is highly abundant, whereas that of Kv1.3, Kv1.4, Kv1.5, and Kv1.6 is present at lower levels in the DRG [60]. A higher level of Kv1.2 protein is also detected in DRG. Approximately 70% DRG neurons are positive for Kv1.2 [45,46,54]. Although an earlier study reported that Kv1.2 was expressed in small DRG neurons [58], subsequent reports from different groups revealed that Kv1.2 was distributed predominantly in medium and large DRG neurons [45,46,54]. In neuron profiles, approximately 72% of Kv1.2-positive neurons are large, 19% are medium, and 9% are small [46]. This observation was further confirmed by the use of the double immunohistochemical labeling for Kv1.2 and specific cytochemical markers. Most (80.3%) Kv1.2 co-localizes with NF-200, a marker for the myelinated A-fibers and corresponding large and medium DRG neurons. Some (11.1%) Kv1.2 co-localizes with P2X3, a marker for small DRG non-peptidergic neurons and some (10.7%) Kv1.2 co-localizes with CGRP, a marker for small DRG peptidergic neurons [46]. Given that nerve injury-induced increase in spontaneous ectopic activity is found primarily in injured myelinated afferents [61-63], the unique subpopulation distribution of Kv1.2 in DRG large and medium neurons indicates possible role in neuropathic pain genesis following Kv1.2 downregulation.

Accumulating evidence showed a time-dependent decrease in the expression of Kv1.2 mRNA and protein in the injured (but not intact) DRG neurons following peripheral nerve injury [45,46,54-59]. The ratio of ipsilateral to contralateral Kv1.2 mRNA in L_5_ DRGs after unilateral L_5_ spinal nerve ligation (SNL) was decreased by 57% on day 3, 86% on day 7, and 82% on day 14 post-SNL compared to the corresponding time points in sham groups [45]. The level of Kv1.2 protein in the ipsilateral L_5_ DRG was reduced by 32% on day 3, 68% on day 7, and 78% on day 14 post-SNL compared to the corresponding time points in sham groups [45]. Consistently, the number of Kv1.2-positive neurons in the ipsilateral L_5_ DRG was diminished by 25% on day 3, 85% on day 7, and 52% on day 14 post-SNL compared to the number at the corresponding time points in the contralateral DRGs of the sham groups [46]. These reductions occurred predominantly in large and medium DRG neurons. No dramatic changes in amounts of Kv1.2 mRNA and protein and in the number of Kv1.2-positive neurons were seen in the contralateral L_5_ DRG, the ipsilateral L_4_ DRG, or contralateral L_4_ DRG during the observation period. Nerve injury-induced Kv1.2 downregulation in the injured DRG were also observed following sciatic nerve axotomy or chronic constriction injury [45,46].

### P2.3. Contribution of MZF1-triggered Kv1.2 antisense RNA to nerve injury-induced DRG Kv1.2 downregulation

An endogenous Kv1.2 antisense (Kv1.2 AS) RNA, a long noncoding RNA, was recently identified. It functions as a biologically active regulator for Kv1.2 mRNA and protein [45]. Overexpression of full-length Kv1.2 AS RNA in cultured HEK293T cells or in primary cultured DRG neurons significantly knocked down Kv1.2 mRNA and protein, but not the mRNAs and proteins of Kv1.1, Kv1.4, and Nav1.8 [45]. In *in vivo* experiments, Kv1.2 AS RNA overexpression time-dependently reduced Kv1.2 mRNA in the DRG [45]. No changes were observed in the expression of Kv1.1, Kv1.4 or Nav1.8 at the levels of mRNA or protein in the DRGs injected with AAV-Kcna2 AS RNA [45]. The evidence indicates that Kv1.2 AS RNA specifically and selectively targets Kv1.2 RNA and protein.

Kv1.2 AS RNA was upregulated in the injured DRG following peripheral nerve injury. The ratio of ipsilateral to contralateral Kv1.2 AS RNA in L_5_ DRGs increased by 1.4-fold on day 3, 3.3-fold on day 7, and 3.3-fold on day 14 post-SNL compared to the corresponding time points in sham groups [45]. Consistently, the number of Kv1.2 AS RNA-labeled neurons in the ipsilateral L_5_ DRGs increased by 1.5-fold on day 3, 2.8-fold on day 7, and 3-fold on day 14 after SNL compared to the corresponding time points in the contralateral L_5_ DRGs [45]. Moreover, the ratios of Kv1.2 AS RNA to Kv1.2 mRNA increased, particularly in individual medium and large DRG neurons after SNL as demonstrated using single-cell real-time RT-PCR analysis [45]. An increase in Kv1.2 AS RNA was also observed in the injured DRG after sciatic nerve axotomy or chronic constriction injury [45].

**Figure 1:**
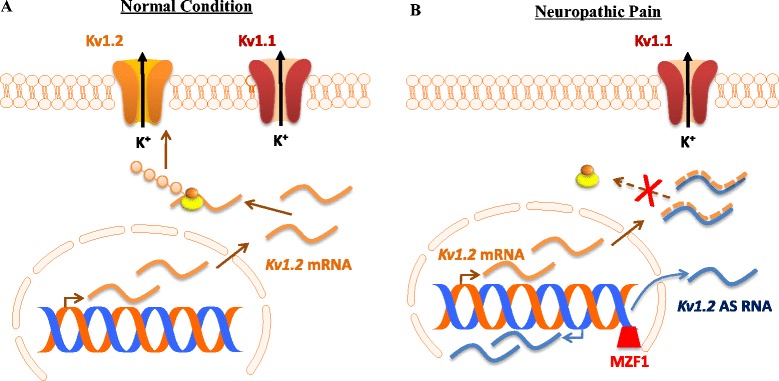
Nerve injury-induced Kv1.2 downregulation triggered by myeloid zinc finger protein 1 (MZF1)-mediated Kv1.2 antisense (AS) RNA expression in the injured dorsal root ganglion (DRG). **(A)** Under normal conditions, Kv1.2 mRNA that is transcribed from the genome is translated into Kv1.2 protein, resulting in normal expression of Kv1.2 channel at DRG neuronal membrane. **(B)** Under neuropathic pain conditions, peripheral nerve injury promotes the expression of the transcription factor MZF1 in DRG. The increased MZF1 binds to the promoter region of Kv1.2 AS RNA gene and triggers its expression. The latter specifically and selectively inhibits the expression of Kv1.2 mRNA via extensive overlap of their complementary regions, leading to a reduction in the membrane expression of Kv1.2 only, not other Kv subunits (e.g., Kv1.1), in the DRG neurons.

Myeloid zinc finger gene 1 (MZF1), a transcription factor, triggers the activation of Kv1.2 AS RNA gene expression in the injured DRG following peripheral nerve injury. Kv1.2 AS RNA gene promoter contains a consensus MZF1-binding motif (-161 to -154). Once bound to this motif, MZF1 promots transcription of target genes [64,65]. In DRG, MZF1 was reported to bind to this motif on the Kv1.2 AS gene promoter [45]. SNL time-dependently increased MZF1 expression and its binding activity in the injured DRG [45]. Moreover, MZF1 directly promoted Kv1.2 AS gene transcriptional activity and was co-expressed with Kv1.2 AS RNA in DRG neurons [45]. It is very likely that nerve injury-induced downregulation of DRG Kv1.2 mRNA is attributed to MZF1-triggered upregulation of DRG Kv1.2 AS RNA under neuropathic pain conditions (Figure 1). It is worth noting that the nerve injury-induced decrease in Kv1.2 mRNA and protein might be caused by other mechanisms at transcriptional and translational levels. These mechanisms will be addressed in future studies.

### P2.4. AAV mediated transfer of Kv1.2 sense RNA into the injured DRG, a strategy for neuropathic pain treatment

**Figure 2:**
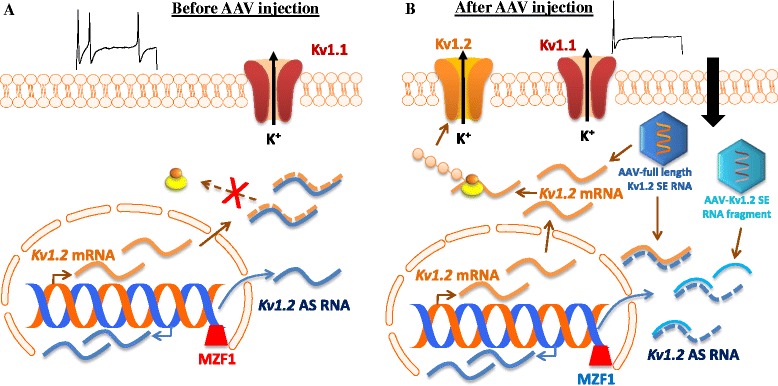
Adeno-associated virus (AAV) mediated transfer of Kv1.2 sense RNA for the reduction of DRG neuronal excitability. **(A)** Before AAV injection into the DRG of rats with peripheral nerve injury, a nerve injury-induced increase in DRG Kv1.2 AS RNA triggered by MZF1 knocks down expression of Kv1.2 mRNA and protein, resulting in an increase in DRG neuronal excitability under neuropathic pain conditions. **(B)** After AAV injection into the DRG of rats with peripheral nerve injury, AAV mediated transfer of full length Kv1.2 sense (SE) RNA rescues nerve injury-induced DRG Kv1.2 downregulation at the DRG neuronal membrane through not only its direct translation into Kv1.2 protein but also its indirect blockage of nerve injury-induced increase in Kv1.2 AS RNA expression via extensive overlap of their complementary regions. AAV mediated transfer of Kv1.2 SE RNA fragment (-311 to +40) also rescues nerve injury-induced DRG Kv1.2 downregulation through its blockage of nerve injury-induced increase in Kv1.2 AS RNA expression via partial overlap of their complementary regions, although this RNA fragment cannot be translated into Kv1.2 protein. Maintaining normal Kv1.2 expression at DRG neuronal membrane reduces nerve injury-induced neuronal hyperexcitability at DRG neurons and consequently decreases spinal central sensitization, resulting in neuropathic relief.

Nerve injury-induced downregulation of DRG Kv1.2 may contribute to neuropathic pain development and maintenance. Mimicking nerve injury-induced DRG Kv1.2 downregulation reduced total Kv current, depolarized the resting membrane potential, decreased the current threshold for activation of action potentials, and increased the number of action potentials in large and medium DRG neurons [45]. Rescuing the SNL-induced downregulation of DRG Kv1.2 by blocking SNL-induced upregulation of DRG Kv1.2 AS RNA through microinjection of a AAV5 Kv1.2 sense RNA fragment (-311 to +40) into the injured DRG attenuated the induction and maintenance of SNL-induced mechanical allodynia, cold hyperalgesia and thermal hyperalgesia [45]. Moreover, overexpressing DRG Kv1.2 in the injured DRG through microinjection of AAV5 full length Kv1.2 sense RNA rescued SNL-induced downregulation of DRG Kv1.2 mRNA and protein and mitigated SNL-induced mechanical allodynia, thermal hyperalgesia, and cold hyperalgesia during both the development and maintenance phases [46]. Overexpressing DRG Kv1.2 sense RNA in the injured DRG also degraded the DRG Kv1.2 AS RNA induced by SNL through the extensive overlap of their complementary regions (Figure 2) [46]. The evidence suggests that DRG Kv1.2 is a key player in neuropathic pain genesis. Given that nerve injury-induced abnormal ectopic activity in the injured myelinated afferents is considered to be a contributor of neuropathic pain genesis [61-63], rescuing Kv1.2 downregulation through AAV mediated transfer of these two Kv1.2 sense RNAs may maintain normal resting membrane potential and diminished ectopic activity in the injured DRG neurons, resulting in reduction of primary afferent transmitter release and attenuation of spinal central sensitization formation and neuropathic pain (Figure 2). Since AAV mediated transfer of neither Kv1.2 sense RNA fragment nor full length Kv1.2 sense RNA affected basal nociceptive response, capsaicin-induced acute pain, and locomotor function [45,46], the strategy of AAV mediated transfer of Kv1.2 sense RNA may have clinical implication in neuropathic pain treatment.

### P2.5. Conclusion

The evidence described above suggests that Kv1.2 is a critical subunit in the Kv channel family of DRG neurons during neuropathic pain development and maintenance. Kv1.2 may be a new target for this disorder. Given that specific and selective Kv1.2 activators are unavailable commercially, AAV-mediated transfer of Kv1.2 sense RNA fragment or full length into the DRGs offers the possibility of long-term rescue of nerve injury-induced DRG Kv1.2 downregulation under neuropathic pain conditions, although this transfer takes 3-4 weeks for Kv1.2 sense RNA expression. Such gene transfer to the peripheral primary sensory neurons may have a potential clinical application for the prevention and treatment of neuropathic pain.

## Part 3. Genetic therapy of chronic pain in a rat model of post herpetic neuralgia induced by varicella zoster virus employing herpes simplex virus vectors expressing enkephalin and the TNFα soluble receptor

(Jean-Marc G Guedon, Joseph C Glorioso, Paul R Kinchington, William F Goins^,^ Corresponding authors, Paul R Kinchington, Ph.D.kinchingtonp@upmc.edu, William F Goins, Ph.D. goins@pitt.edu)

*Post herpetic neuralgia (PHN) is a debilitating, difficult-to-treat and poorly understood pain state that may follow herpes zoster, which is caused by the reactivation of varicella zoster virus (VZV) from neuronal latency. To shed light on the mechanisms underlying PHN and to test pre-clinical treatments, a rat model has been developed in which VZV inoculation at the footpad induces prolonged nocifensive behaviors that mimic mechanical allodynia and thermal hypersensitivity seen in human PHN patients. Various analgesics have been evaluated in the model, including agents currently used to treat PHN (gabapentin, amitriptyline, morphine, etc) as well as novel therapeutic candidates. One disadvantage of most of these therapies is that the pain relief is often only temporary and may require frequent or continuous dosing, or employ unfavorable routes of administration. To seek prolonged analgesia, we employed replication-deficient herpes simplex virus (HSV) based vectors expressing the natural opioid, preproenkephalin (vHPPE). Peripheral administration of vHPPE induced a dose-dependent prolonged duration of relief of VZV-induced hypersensitivity, provided extended relief upon re-inoculation, and could prevent nocifensive behaviors from developing when administered prophylactically. HSV-based vHPPE vectors displayed promise in a human Phase-I trial for the treatment of bone cancer pain, supporting their potential use in extended relief of human PHN. A second treatment strategy was based on gene array analyses of the dorsal root ganglia (DRG) from rats exhibiting VZV-induced nocifensive behaviors, which revealed up-regulation of the TNF receptor associated death domain TRADD and TNFrsf21 the TNF receptor superfamily member also known as death receptor 6 (DR6) that interacts with TRADD via its death domain. This specific change in expression suggested immune mediated mechanisms contribute to VZV-induced pain in the rat model. In agreement, administration of replication-deficient HSV vector expressing the human soluble TNFα receptor (sTNFR) was found to reduce VZV-induced hypersensitivity in a prolonged manner. These data suggest a role for TNFα in VZV-induced pain.*

### Keywords

*Post Herpetic Neuralgia, Enkephalin, Tumor Necrosis Factor, Rat Model of PHN, Varicella Zoster Virus*

### P3.1.VZV, Zoster and PHN

The human herpesvirus varicella zoster virus (VZV) causes varicella (chickenpox) upon primary infection and then establishes a latent state within neurons of sensory and autonomic ganglia. Approximately one third of the VZV-infected patient population will subsequently develop the reactivated disease zoster (shingles), usually after age 60 or following immune compromise from disease or iatrogenic cause. A host of neurological problems are associated with zoster, including numbness, itch, ischemia, vasculopathies, myelitis and ophthalmoplegia, but by far the most common complication is pain [66-68]. Up to 90% of zoster patients are prescribed pain relieving medication, particularly to mitigate zoster associated pain (ZAP) that occurs before, during or shortly after the appearance of zoster skin lesions. Treatment for ZAP is effective with antiviral and/or NSAID administration [69,70], particularly when initiated early. However, 33% of zoster patients develop a chronic, debilitating and difficult to treat pain state known as post herpetic neuralgia (PHN), defined as chronic pain lasting longer than 30 days. PHN pain is described as stimulus-independent (spontaneous) pain, stimulus-evoked pain (allodynia), and evoked/non-evoked “stabbing” pain [67]. The pain of PHN may be so debilitating as to lead to secondary consequences such as depression, withdrawal from society, and a dramatic decrease in patient quality of life [67,71,72]. While PHN patients have a variety of available treatment options, there is no “silver bullet” that alleviates pain in all patients. Indeed, a significant fraction of PHN patients will not experience even partial relief from any current treatment options, which include opiate based treatments such as morphine; anti-convulsants such as amitryptiline, pregabalin, and gabapentin; topical treatments including capsaicin and lidocaine patches; and more unconventional treatments such as ganglionectomy and large-area skin replacement [73]. Many drug treatments induce unwanted off-target whole-body side effects, and some lead to tolerance, abuse and addiction [67,69].

### P3.2. Inflammatory components of pain

Inflammatory components contributing to pain have long been appreciated for their role in nociception. There are three pro-inflammatory cytokines that have been associated with neuropathic pain, namely tumor necrosis factor alpha (TNFα), interleukin-1 (IL-1), and interleukin-6 (IL-6) [74-79]. As a consequence, use of broad anti-inflammatory treatments such as NSAIDs are popular first-line choices for the treatment of inflammatory pain, including ZAP associated with herpes zoster. NSAIDs reduce inflammation involved in pain initiation or maintenance. However, NSAIDs and similar cyclooxygenase targeting anti-inflammatory drugs frequently have little effect on pain associated with PHN, suggesting PHN pain is more complicated than just an inflammatory process. Indeed, the efficacy of anti-cytokine immune biologics to alter pain in PHN patients has proven confusing. While antibodies that interfere with TNFα such as Etanercept, Adalimumab and Infliximab have been successfully employed to relive pain in patients with rheumatoid arthritis, inflammatory bowel disease and other immune complications, reports vary widely for treatment of PHN pain with some showing efficacy without complications in patients with herpes zoster (HZ), ZAP or PHN, while others suggest that the severity and risk of PHN are dramatically increased [80-82].

In an effort to gain a better understanding of the role of inflammation in pain, rodent pain models have been extensively employed to examine how inflammation initiates and/or amplifies pain signaling. Various inflammatory pain models exist where administration of inflammatory mediators, including TNFα, contribute to pain and blockade of these mediators has provided substantial pain relief [83-86]. TNFα, as a major mediator of inflammation, is up-regulated in many chronic pain models, including chronic constriction injury (CCI), carrageenan- and zymosan-induced pain, and spinal nerve transection [87-90]. Following injury, one can readily detect increased levels of TNFα in both microglia and astrocytes within the dorsal horn of the spinal cord where afferents project from the location of the injury [91], suggesting that release of the cytokine into the cord plays a role in nociception. Moreover, activated mast cells, macrophages, and neutrophils also produce cytokines including TNFα [92-94]. TNFα treatments can lead to peripheral neuropathy [95] in cancer patients, and direct injection of TNFα in normal naïve animals results in pain-like behaviors similar to those described in human patients with rheumatoid arthritis (RA) and other inflammatory diseases [81,96,97]. TNFα is thought to be one of the major players involved in neuro-immune activation of pain signaling within the dorsal horn of the spinal cord [98,99], but its role in PHN has not been determined. Below, we present preclinical data that implicates TNFα and its downstream effectors are associated with VZV-induced chronic hypersensitivity in rats.

### P3.3. The rat model of VZV-induced chronic pain

A preclinical model of chronic VZV-induced pain in rats, first described by Fleetwood-Walker et al [100], has been the subject of recent reviews and will only be summarized here [101,102]. The model is initiated with footpad inoculation of cell-associated VZV, that consists of VZV-infected cells rather than virus that has been purified away from the infected cells, with uninfected cells used as the negative control. Cell-associated VZV is used because cell-free VZV cannot be prepared to the titers required to induce pain, due to the highly cell-associated nature of VZV in culture. Rats subsequently develop strong hypersensitivity within one week to mechanical stimuli and to a lesser extent to thermal (heat but not cold) stimuli, as well as develop anxiety behaviors in open field test paradigms [101,103-109]. The mechanical hypersensitivity observed in the rodent model reflects the intense allodynia experienced by more than 70% of all human zoster patients [110,111], while thermal-induced pain is also seen in a smaller PHN patient population [112]. In our hands, mechanical hypersensitivity is more robust and longer lasting compared to thermal responses [104]. Hypersensitivity in animals correlate with VZV presence in the corresponding DRG, and the expression of a limited subset of viral genes and proteins in both large and small diameter neurons, defined by positive staining for neurofilament 200 (NF200), neuropeptide-Y (NPY) and peripherin. VZV infection also correlates with ganglionic changes in expression of galanin, activating transcription factor 3 (ATF-3), the α2∂1 calcium channel, and voltage gated sodium channels Na_v_1.3 and 1.8, suggesting a neuropathic basis for the VZV-induced pain [103]. Animals infected with VZV still develop pain when treated with the viral replication inhibitors acyclovir and valacyclovir, suggesting pain develops without the need for viral DNA replication [106,109]. This mirrors human PHN patients, who largely see no effective pain relief from acyclovir when taken after 72 hours from the beginning of zoster [69,113]. However, viral infectivity and/or *de novo* VZV transcription is necessary for the onset of nocifensive behaviors in rats, as UV-inactivated VZV failed to induce hypersensitivity to either mechanical or thermal stimuli [104]. It is important to note that a major difference between the rat model and human PHN is the trigger of chronic hypersensitivity occurs upon initial infection in the rat, while in humans, pain is only associated with reactivation of VZV from latency within sensory neurons, and is almost never associated with primary infection. As yet, there is no reliable animal model of VZV reactivation, so this issue remains to be resolved. In conclusion, the rat PHN model is a reproducible and reliable model that reflects many of the clinical conditions seen in human PHN patients, and thus provides the only platform to address mechanisms underlying VZV-induced pain.

There has been extensive use of the model to test therapeutics, both those known to be efficacious in treating human PHN, as well as novel investigational agents and compounds. Due to the nature of the host immune response involved in zoster and ZAP, early studies evaluated the use of NSAIDs to reduce the host inflammatory response, with conflicting reports. While systemically applied ibuprofen reduced both mechanical allodynia (MA) and hypersensitivity [105], another group failed to see any relief using the anti-inflammatory NSAID diclofenac [103]. However, in general, drugs used in the rat model usually replicate the efficacy for the agent in treating the human PHN condition. Agents shown to have some effect include: (i) morphine, (ii) gabapentin, (iii) the tricyclic antidepressant amytriptiline, and (iv) sodium channel blockers mexiletine and lamotrigine [103,105,108]. Novel agents that have been evaluated and are also somewhat efficacious include: (i) astrocyte toxin (L-α-aminoadipate), (ii) iNOS inhibitors (L-N6-(1-iminoethyl)-lysine), (iii) nitric oxide scavengers (2-Phenyl-4,4,5,5-tetramethylimidazoline-1-oxyl 3-oxide), (iv) IL-1 receptor antagonist, (v) the cytokine inhibitor (Pentoxifyline), (vi) the NMDA receptor antagonist ((2*R*)-amino-5-phosphonovaleric acid; (2*R*)-amino-5-phosphonopentanoate and 3-((R)-2-Carboxypiperazin-4-yl)-propyl-1-phosphonic acid),(vii) and the non-competitive NMDA receptor antagonist (Dizocilpine) [103,105,109]. The relief obtained by these treatments is frequently short-lived, lasting only hours or for the duration of the drug administration. Furthermore, many of the evaluated treatments have administration routes that are prohibitive in humans, and in many instances, do not provide pain relief after multiple applications. This supports the need for more effective and prolonged therapeutic strategies.

### P3.4. Gene therapy using herpes simplex virus (HSV) replication-deficient vectors to alleviate hypersensitivity in the rat model of VZV-induced PHN

While the rat model of PHN has been employed for evaluation of several drug therapies, it has only recently been used to evaluate gene delivery and expression therapy approaches. HSV vectors are uniquely positioned for the treatment of pain, as HSV vectors; (i) effectively target and enter peripheral innervating neurons (ii) can be injected easily into peripheral tissues and traffic to ganglia innervating the dermatome that was injected; (iii) are capable of delivering and expressing large genes and even multiple genes; (iv) can be easily manipulated and grown to high titers *in vitro;* and (v) have shown safety in humans for the treatment of chronic pain without the generation of any adverse events [10]. HSV vectors are rendered replication defective by deletion of several genes critical to the control of viral gene expression (including ICP4 and ICP27), and are grown on cell lines that express such genes *in trans.* While an extensive review of HSV vectors and pain is beyond the scope of this article (see [114]), it should be pointed out that HSV based vector delivery and expression at the ganglia has been employed to treat a variety of chronic pain conditions in preclinical models through the expression of several pain relieving genes including: (i) glial cell derived neurotrophic factor (GDNF), (ii) vascular endothelial growth factor (VEGF), (iii) erythropoietin (EPO), (iv) the glutamic acid decarboxylase 67kD isoform (GAD67), (v) the alpha-1 subunit of the glycine receptor (GlyRα1), and (vi) dominant negative form of protein kinase C epsilon (dnPKCε); as well as antisense genes for (vii) voltage gated sodium channel 1.7 (Nav1.7) or (viii) the α subunit of the GABA receptor (GABA-B1αR), (ix) the mu-opioid receptor (μ-OR), (x) the calcitonin gene related peptide (CGRP), and (xi) enkephalin. These vectors have been evaluated in pain models including: pain associated with pancreatitis, formalin injection, spinal nerve ligation, complete Freund’s adjuvant (CFA)-induced arthritis, chronic constriction injury (CCI), bone cancer pain, pertussis toxin (PTx)-induced pain, and a bladder nociception model. HSV based vectors have also been used to express modulators of inflammatory processes, such as interleukin 4 (IL-4), interleukin 10 (IL-10), Iκβ or the soluble form of the TNFα receptor (sTNFR) [12,13,115-122]. Many of these vector-mediated immune therapies result in reduced levels of pro-inflammatory cytokines such as TNFα, IL-1β, IL-6, PGE2, spinal cord c-Fos and even phosphorylated p38 MAPK, demonstrating that their decreased expression results in reduced nocifensive behaviors in pain models. A Phase-I clinical trial to treat cancer pain with HSV based vectors has established the clinical safety and lack of problems associated with the use of these vectors [10] as well as showing efficacy at the highest dose that was transient lasting for 2-4 weeks.

**Figure 3:**
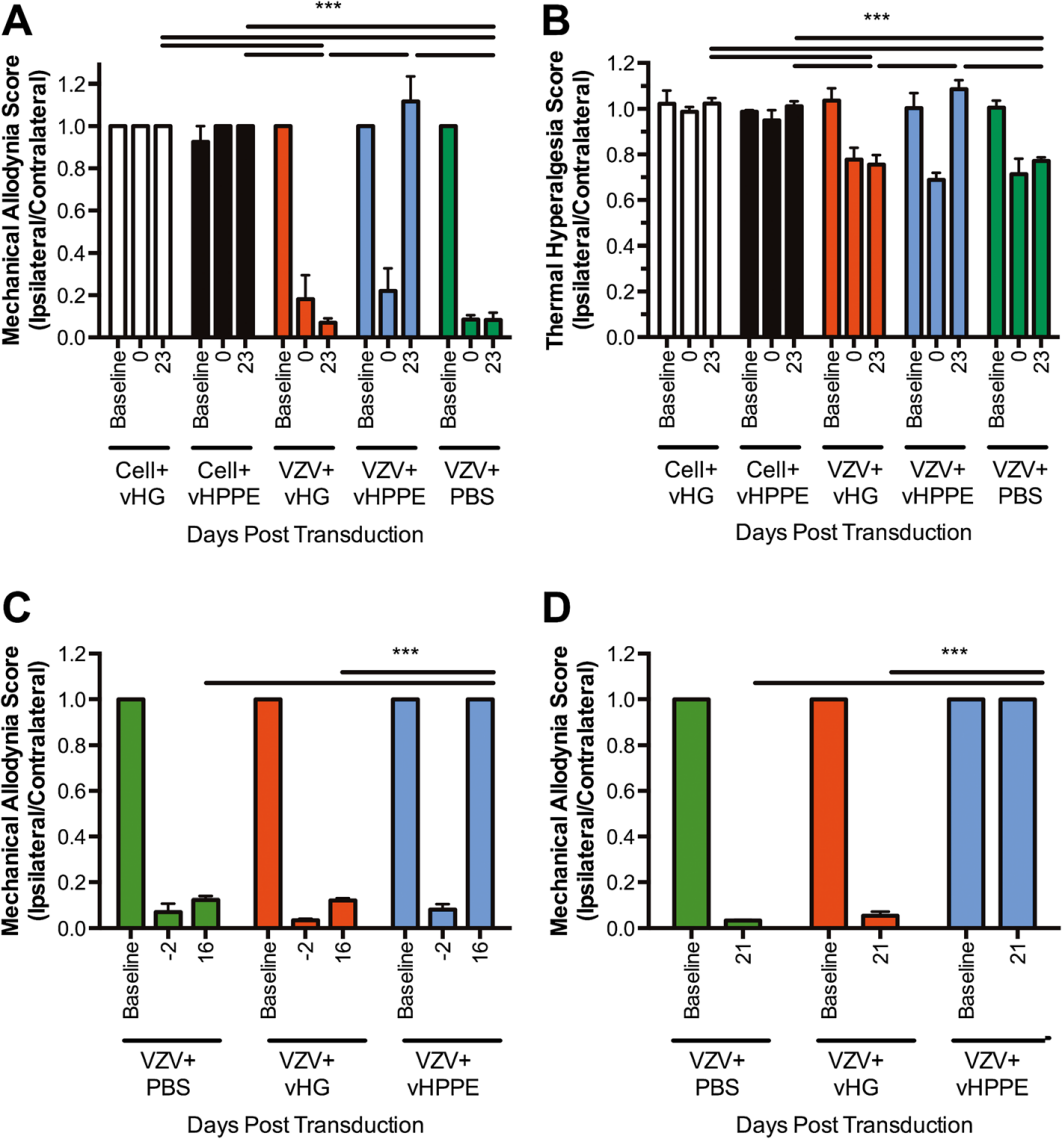
Herpes simplex vector-mediated enkephalin expression alleviates VZV-induced nocifensive behaviors in the rat PHN model. Animals (n = 5) were injected with PBS vehicle, 10^8^ or 10^4^pfu of HSV vector 19 days after VZV or control cell inoculation. Animals received either control vector (vHG) or vector expressing human preproenkephalin (vHPPE). Hypersensitivity was measured to **(A)** von Frey filament stimulation or **(B)** thermal sensitivity using a Hargreaves apparatus. **(C)** Mechanical hypersensitivity following 10^4^pfu (at day 0) of vehicle, vHG or vHPPE. **(D)** Prophylactic vector administration, with animals receiving VZV at 7 days post vector inoculation. Data is presented as a ratio of ipsilateral to contralateral responses Mean + SEM. All times listed refer to days post HSV sTNFR-expression vector inoculation/transduction.One way ANOVA with Bonferroni post comparing all columns at each timepoint (*** = p < 0.001).

While the rat PHN model has been used to evaluate effects of various drug therapies [103,105,108], gene therapy approaches to modify VZV-induced pain had not been evaluated until recently. We evaluated two different HSV-1 gene therapy vectors in the rat PHN model. The first was a vector expressing human preproenkephalin (hPPE) in which expression was driven by the hCMV immediate early promoter and transcriptionally terminated using a bovine growth hormone polyadenylation signal (BGHpA). The vector was shown to alleviate hypersensitivity in a dose-dependent manner, with the highest dose of 10^8^ plaque forming units of virus (pfu) resulting in complete long-term alleviation of VZV-induced mechanical and thermal hypersensitivity (Figure 3; [104]). While inoculation with a lower dose (10^4^ pfu) resulted in only 10 days of relief, hypersensitivity was reduced following re-dosing of those animals with 10^8^ pfu of vHPPE. This suggests reapplication of vHPPE could reduce hypersensitivity in situations where analgesic relief of VZV-induced pain is outlived by the long-term nature of pain in PHN patients, which may last months to years. It further suggests that first application of HSV vectors did not induce adaptive host immune responses that could effectively block subsequent re-dosing and the resulting vector-mediated relief. We also established prophylactic treatment with 10^8^ pfu of vHPPE prior to VZV inoculation could prevent the development nocifensive behaviors. This is highly significant to clinical PHN as prodromal signs often precede zoster, and vectors could in theory be applied to any patient at the zoster lesion stage prior to the development of chronic pain. The vHPPE effects on nociception were most likely acting at the level of the ganglion, since peripherally administered DAMGO, a μ-opioid receptor agonist, did not alleviate VZV-induced mechanical hypersensitivity, nor did peripherally administered Naloxone, a opioid receptor antagonist, block vHPPE-mediated pain relief. These results clearly suggest translational use of gene therapy approaches for long-lasting treatment of VZV-induced hypersensitivity.

**Figure 4:**
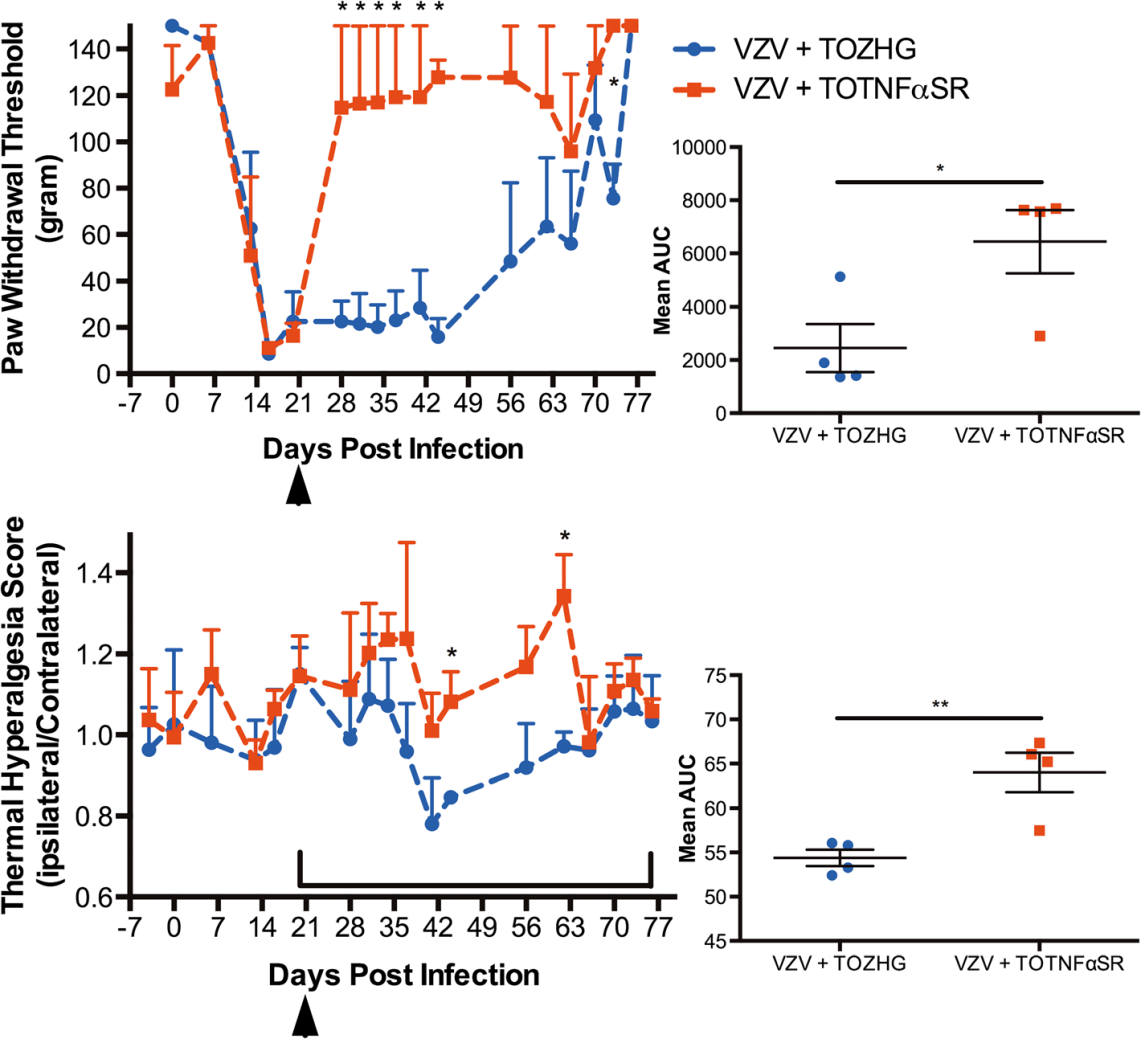
Alleviation of VZV-induced hypersensitive nocifensive behaviors by administration of TNFα soluble receptor (sTNFR) expressed from an HSV vector. All animals (n = 4) were infected with VZV at day-0. At 21 days post VZV infection (denoted by arrowheads), animals were inoculated with either sTNFR vector (TNFasR) or control vector (T0ZHG). Animals were monitored for hypersensitivity to (top panel) mechanical (gram weight) and (bottom panel) thermal sensitivity (ratio of ipsilateral/contralateral paw withdrawal latencies) and the data is plotted as Mean + SEM. Each time-point was compared by two-tailed T-test (* = p < 0.05). Mean area under the curve was calculated for the region represented by brackets for each animal and compared by two-tailed T-test (* = p < 0.05, ** = p < 0.01) with the Mean ± SEM plotted.

VZV infection in the rat PHN model clearly induces changes at the DRG that correlate with the development of the pain response. Previous work had shown up-regulation of the pain-associated genes NPY and ATF-3 [103]. An Affymetrix rat gene array analysis followed by RT-PCR validation of up- and down-regulated mRNAs revealed the up-regulation of expression of tumor necrosis factor superfamily associated death domains (TRADD and TNFsrf21) at 10- days post infection (dpi) with the HSV vector expressing sTNFR. These genes are involved in the TNFα signaling pathway, which has been shown to be up-regulated in both the rat PHN model (unpublished observations) and the *in vivo* SCIDhu VZV models [123]. Since TNFF061, IL-1ß and IL-6 are the main pro-inflammatory cytokines secreted by various activated immune and glial cells in other models of inflammatory and neuropathic pain [83,85,86,98,99,124], this suggested a potential role for TNFα signaling in VZV-induced pain and provided us a new target for intervention. To clarify the role of TNFα in VZV-induced pain, we tested HSV vector-mediated expression of the human TNFα soluble receptor (sTNFR) for its effects on VZV-induced hypersensitivity. Such vectors have been shown to be effective in other pain models including the rat L5 spinal nerve ligation (SNL) model [121], the T11-T12 laminectomy spinal cord injury (SCI) model [122], the resiniferatoxin (RTx)-induced model of bladder nociception [118] as well as HIV gp120-induced neuropathic pain [125]. Animals inoculated with VZV (pOka strain) at day 0 rapidly established robust indicators of hypersensitivity, and were treated at day 21 with either control HSV vector (T0ZHG) or HSV expressing sTNFR (T0TNFαsR) (Figure 4) at a dose of 10^8^ pfu. Animals inoculated with sTNFR showed a rapid and sustained decrease in hypersensitivity within days following T0TNFαsR administration, while control vector inoculated animals did not. Mechanical paw withdrawal thresholds increased as early as 7 days after HSV sTNFR-expressing vector inoculation and lasted until 23 days post HSV vector inoculation (Figure 4) or 44 days post the introduction of VZV-infected cells. However, after 35 days post HSV vector treatment the mechanical hypersensitivity began to spontaneously resolve in VZV-infected and untreated animals, as we and others have seen previously [100,103-106,109]. Compared to the effects of vector-mediated sTNFR on mechanical pain, thermal relief took longer to respond to sTNFR HSV gene therapy but the response lasted about the same length of time. With the thermal pain response relief started at 23 days post HSV sTNFR-expressing vector inoculation (44 days post the introduction of VZV-infected cells) and lasted till 41-dpi after HSV vector injection (Figure 4). Interestingly mean area under the curve plots suggested that not all animals responded to treatment. Taken together, it appears that TNFα plays an important role in VZV-induced pain and opens up a new target for analgesic relief of VZV-induced pain. It suggests that PHN may be predicted to respond to biologics that target TNFα, a key inflammatory component. However, some severe complications due to reactivated VZV seen in RA, inflammatory bowel disease (IBD), Crohn’s disease, ankylosing spondylitis, plaque psoriasis and psoriatic arthritis patients receiving anti-TNF treatments to reduce the host immune response suggests that whole-body treatment by anti-TNF injectable biologics may not be an optimal therapy for all PHN patients [82,126-132].

### P3.5. Concluding remark

Genetic delivery and expression of pain modulators is a novel and durable treatment strategy for seeking analgesia of VZV-induced pain that has potential for application to PHN in patients. The strategy should be considered an improvement over drug treatments, since the vector is targeted to a specific area, has long-term effects after a single dose, and can in theory be reapplied to give sustained analgesia, which would be needed for human PHN that sometimes last for months to years. In addition to delivery of the tonal modulator enkephalin, we now have established a second strategy which relies on expression of the anti-inflammatory sTNFR from vector-transduced ganglia. Targeted expression of sTNFR from HSV vectors may help circumvent issues with systemic administration of anti-TNF drugs to treat PHN. Other gene delivery vectors such as Adenovirus, Adeno-associated virus (AAV) and lentivirus vectors have been used in other models to treat chronic pain however, due to the natural biology of HSV it displays several fold greater efficiencies in transducing PNS neurons [50], the target tissue for pain gene therapies, and is the only vector used in human pain gene therapy trials [49]. Cytokines and their role in chronic neuropathic pain have long been appreciated, especially TNFα, who’s administration results in pain states in rodents [79,83,124,133] and has long been thought to be an important mediator of neuropathic pain in humans. It remains to be determined what the source of TNFα is and what cells are generating it in response to VZV infection, but other groups have shown that glia (microglia, astrocytes and even Schwann cells) are responsible for TNFα expression during other chronic pain conditions [133]. As such this may hold true for the VZV-PHN rat model as suggested by Zhang and colleagues [109].

## Part 4. Viral vector-mediated overexpression of anti-inflammatory cytokines for chronic pain and morphine tolerance/withdrawal

(Xuexing Zheng, Ching-Hang Liu, Shue Liu, Shuanglin Hao, Corresponding address: Shuanglin Hao, shao@med.miami.edu)

*Chronic pain has been described as the result of dysfunctional activity of glia and neurons. Development of tolerance and dependence is a major problem associated with opioid treatment of chronic pain. Previous evidence suggests similar cellular mechanisms in pain hypersensitivity and morphine tolerance/withdrawal. Proinflammatory cytokines are involved in the development and maintenance of both neuropathic pain and chronic morphine tolerance/withdrawal. In this article, we review viral vector-mediated gene transfer of anti-inflammatory cytokines in rodent models of neuropathic pain and chronic morphine tolerance/withdrawal, which support the use of the gene therapy approach in the clinic.*

### Keywords

*Gene transfer, anti-inflammatory cytokines, chronic pain, morphine tolerance/withdrawal*

### P4.1. Introduction

Chronic pain is a maladaptive, pathologic and persistent condition that results in marked decrease in the quality of life and secondary symptoms such as anxiety and depression. One third of the adults in the United States [134,135] and one fifth of the adults in Europe [136] are affected by chronic pain. Many conventional agents utilized as pharmacological therapy for chronic pain are not very effective for providing satisfactory analgesia. The drawback of most common systemic administration of drugs is the difficulty in selectively targeting specific pain-related pathways in the nervous system. Off-target effects of drugs are particularly problematic in attempts to treat diseases, because of the wide anatomic distribution of most drugs’ targets [2]. Opioids and opioid-derivatives (especially morphine) are most widely used drugs to treat moderate to severe chronic pain. However, chronic opioid administration tends to induce tolerance, hyperalgesia, and withdrawal, which hinder the efficacy of opioid treatment [137-140].

Viral vector-mediated gene therapy is used to persistently deliver short-lived bioactive molecules to restricted anatomic locations. The local production of neurotransmitter/neuropeptide achieved by viral vector mediated gene transfer, may be used to achieve desired outcomes; simultaneously, the site-restricted neurotransmitters may avoid unwanted adverse side effects that would otherwise result from activation of the same receptors by a systemically administered drug [141].

Recent studies demonstrate viral-vector mediated therapeutic approaches to focal CNS diseases such as Parkinson’s disease [142] and cancer pain [10] in clinical trials. A wide range of viral vectors including adeno-associated virus (AAV)-based vectors and lentiviral (LV) vectors have been used to express neurotrophic factors or enzymes in specific regions [2]. We and others have previously demonstrated that recombinant herpes simplex virus (HSV)-based vectors delivered by subcutaneous inoculation can be used to express neurotransmitters in the DRG, and to produce a pain-relieving effect in different pain models in rodents [143]. Here, in this review, we mainly focus on the mechanisms related to neuroinflammation shared by both chronic pain and morphine tolerance/withdrawal, and treatments with anti-inflammatory cytokines introduced by viral vectors.

### P4.2. The neurochemical mechanisms of chronic pain and morphine tolerance/withdrawal

#### P4.2.1. Chronic pain

While there are many mechanisms of neuropathic pain that are involved in the central sensitization, three main aspects have been identified: neurotransmitter/neuropeptide-mediated hypersensitivity, loss of tonic inhibitory controls (disinhibition), and glial-neuronal interactions [144]. In the spinal cord dorsal horn, primary afferent C/Aδ fibers release peptides (e.g., substance P/calcitonin-gene related peptide (CGRP), etc.) and excitatory amino acid (glutamate) products following tissue injury [145]. The *N*-methyl-d-aspartate (NMDA) receptors are silent under normal conditions, however, in the setting of nerve injury/tissue damage, increased release of neurotransmitters from primary afferent nociceptors sufficiently depolarizes postsynaptic neurons to activate NMDA receptors in second-order neurons in the spinal cord dorsal horn [145]. The consequential increase in calcium influx can strengthen synaptic connections between nociceptors and spinal cord dorsal horn pain transmission neurons, which, in turn exacerbate responses to noxious stimuli [144]. The main type of inhibitory synaptic transmission in the spinal cord dorsal horn is mediated by γ-aminobutyric acid (GABA) and glycine receptors. Partial nerve injury results in reduced presynaptic GABA release and lower expression of GABA synthesizing enzyme glutamic acid decarboxylase (GAD) [146], which contribute to abnormal pain sensitivity and the phenotypic features of the neuropathic pain syndrome [147,148]. This disinhibition induces non-nociceptive Aβ afferents to engage the pain transmission circuitry, such that normally innocuous stimuli are now perceived as painful [144,149-153]. Peripheral tissue damage, nerve injury or inflammation promotes release of neurotransmitters and neuropeptides from primary afferents. Microglial activation occurs within minutes, but can have long-lasting effects [154]. Activations of at least five major paths including fractalkine, interferon-γ, monocyte chemoattractant protein-1, toll-like receptors, and P2X on microglia, are involved in certain neuropathic nociceptive states [155]. Through glia-glia and neuron-glia crosstalk, the synapse creates a link between glial cell activation and neuronal excitation that may contribute to persistent pain [156]. Activated glia release a host of proinflammatory cytokines, such as tumor necrosis factor alpha (TNFα), interleukin 1 beta (IL-1β), interleukin 6 (IL-6), and other factors (e.g. chemokines), which through their receptors expressed by neurons in the spinal cord dorsal horn, promote the increased excitability, and enhance pain in response to both noxious (hyperalgesia) and innocuous stimulation (allodynia) [144,157-159].

#### P4.2.2. Morphine tolerance/withdrawal

Although many mechanisms have been postulated to explain morphine tolerance, substantial evidence shows that in response to chronic morphine, spinal cord glia (mainly astrocytes and microglia) release pro-inflammatory cytokines (e.g., TNFα, IL-1β, and IL-6) [160,161] and excitatory amino acids, nitric oxide, and prostaglandins [162]. These responses contribute to the development of morphine tolerance and tolerance-associated pain sensitization. Previous evidence suggests similar cellular mechanisms in morphine tolerance and pain hypersensitivity [163,164]. Among pro-inflammatory cytokines, TNFα acts as a crucial initiator of inflammatory reaction. TNFα activates transcription factor AP-1 and/or NF-kB to express adhesion molecules, to stimulate other immune cells, and most importantly, and to produce pro-inflammatory cytokines, such as, IL-1β and TNFα. Both IL-1β and TNFα can activate and become activated by NF-kB, thus forming a positive regulatory loop that amplifies and prolongs inflammatory responses [121]. Neutralizing antibodies against TNF receptors are shown to reduce thermal hyperalgesia and mechanical allodynia [165]. Intrathecal administration of the recombinant TNF soluble receptor (TNFSR) peptide (etanercept) reduces mechanical allodynia and spinal nerve ligation-induced p38 activation [166], and also preserves a significant antinociceptive effect of morphine in morphine-tolerant rats [160]. Chronic morphine infusion increases TNFα, IL-1β, and IL-6 mRNA expression in the spinal cord dorsal horn; inhibition of the proinflammatory cytokine attenuates morphine tolerance; and TNFα inhibitor etanercept reduces proinflammatory cytokines production and microglial activation, thus preserving the antinociceptive effect of morphine [160].

### P4.3. Anti-inflammatory cytokines mediated by gene transfer for chronic pain

Opioid peptides (endorphin [167] and enkephalin [7]), glutamate transporter (GLT-1) [168], inhibitory GABAnergic neuron-related proteins (glutamic acid decarboxylase, GAD65 [169] or GAD67 [170]) and neurotrophic factors (glial cell-line derived neurotrophic factor, GDNF [171]) have been successfully delivered through viral vectors to the nervous system to reduce pain in various neuropathic or inflammatory chronic pain models. Here, we focus on anti-inflammatory cytokines mediated by a viral-vector based gene transfer on chronic pain and morphine tolerance/withdrawal. Viral vectors utilized for gene transfer studies mainly include recombinant adenovirus (AD), AAV, LV, and HSV. Summary of characteristics of viral vectors is shown in Table 1.

**Table 1:** **Summary of characteristics of vital vectors used for pain analgesia and morphine tolerance/dependence**

	**AD**	**AAV**	**LV**	**HSV**
**Wild-type**	DS-DNA	SS-DNA	SS-RNA	DS-DNA
**Genome size**	36 kb	4.7 kb	9.2 kb	152 kb
**Capacity of gene insert**	~7.5 kb	~4.5 kb	~8 kb	~40 kb
**Persistence of gene expression**	days-weeks	months-years	months-years	days-years
**Inoculation**	IT	IT	IT, nerve	SC paw, PAG, bladder wall
**Gene product**	IL-10	IL-10	TNF shRNA, IL-10	IL-4, IL-10, TNFSR
**Pain models**	NP	IP	NP	NP, IP
**M-T/M-W**	M-T			M-T, M-W

DS-DNA, double strain DNA; SS-DNA, single strain DNA; SS-RNA, single strain RNA; IT, intrathecal; SC, subcutaneous injection; NP, neuropathic pain; IP, inflammatory pain; M-T, morphine tolerance; M-W, morphine withdrawal.

#### P4.3.1. Interleukin 4

HSV is an enveloped double-stranded 152 kb DNA virus [172]. Immediately after entry of the viral DNA into the nucleus, expression of five viral immediate-early (IE) genes proceeding in the absence of de novo viral protein synthesis commences [173,174]. Recombinant non-replicating HSV-based vectors are created by deletion of one or more essential IE genes. These recombinants retain the neuronal targeting properties of the wild type virus and can be propagated to high titers on complementing cells but are unable to replicate in animals *in vivo* [3,14,175,176]. Because of efficient transduction and naturally targeted neurons, relatively low titers of HSV vectors are required for therapeutic gene transfer. This strategy is analogous to continuous local infusion [3,177,178]. We have demonstrated the utility of these vectors in preclinical models of pain [3,115].

Interleukin 4 (IL-4) is a prototypical anti-inflammatory cytokine that modulates macrophage activity through global suppression of proinflammatory cytokines [179]. IL-4 also has pleiotropic effects on the development of immune cells and the immune response [180]. The HSV-mediated IL-4 expression in a pain study was carried out in the spinal nerve ligation (SNL) model [12]. Subcutaneous inoculation of the HSV vectors expresses murine IL-4 (S4IL4) in the hind foot transduced lumbar DRG. Immunofluorescent staining showed IL-4 expression in large and small neurons in the DRG 1 week after subcutaneous inoculation of vector S4IL4. Rats inoculated subcutaneously in the foot 1 week after L5 SNL and sacrificed 2 weeks later had 23.7 pg murine IL-4 per DRG, compared to no detectable IL-4 in SHZ-inoculated rats. The HSV vectors do not alter thermal latency or tactile threshold in normal animals, but inoculation of S4IL4 one week after SNL reduced mechanical allodynia for 4-5 weeks and reversed thermal hyperalgesia for 4 weeks [12]. S4IL4 reduced SNL-induced the upregulation of spinal IL-1β, PGE2, and pp38 [12]. Interstitial cystitis/painful bladder syndrome (IC/PBS) is characterized by increased bladder pain and urinary frequency and associated with increased IL-2, IL-6, IL-8, TNFα expression, and lowered levels of IL-4 [120]. In the rat IC/PBS model, IL-4 in the bladder and L6 DRG were evaluated using ELISA at 2 weeks after viral vector injection into the rat bladder wall. In the bladder, a significant difference of IL4 expression was detected between the SHZ and S4IL4 animal groups (0.01 ± 0.01 pg/mgTP vs 5.62 ± 1.64 pg/mgTP). In L6 DRG of S4IL4-injected rats, there was 13.27 ± 3.76 pg/mgTP of murine IL-4, whereas no murine IL-4 was detected in L6 DRG of SHZ-injected rats [120]. The increased IL-4 expression mediated by the HSV vector suppressed bladder inflammatory responses, reduced expression of IL-1β and IL-2, and lowered neutrophil activity in the bladder. Bladder overactivity and nociceptive behavior (freezing scores) was significantly suppressed in the S4IL4 vector-injected group by 47%, compared with the SHZ-treated group (30.25 ± 4.71vs 57.14 ± 6.29) [120]. These two independent studies show that HSV-mediated expression of IL-4 effectively reduces pain-related behaviors.

#### P4.3.2. Interleukin 10

Interleukin 10 (IL-10) was first described as a cytokine synthesis inhibitory factor inhibiting cytokine production by Th1 cells, and reducing NF-kB and macrophage activation [181]. The therapeutic effects are proven by various forms of IL-10 in different neuropathic pain models. A single dose of human recombinant IL-10 directly into the lesion site resulted in the reduction of thermal hyperalgesia and numbers of recruited macrophages and TNFα-positive cells [182]. Acute administration of intrathecal IL-10 protein itself briefly reversed sciatic chronic constriction injury (CCI)-induced mechanical allodynia and thermal hyperalgesia [116]. Repeated intrathecal injections of plasmid DNA encoding interleukin-10, produced prolonged reversal of neuropathic pain [183,184].

AD vectors have a gene carrying capacity of ~7.5 kb [185]. Milligan and colleagues used a replication-defective AD vector containing the cDNA encoding for human-IL10 [116]. Intrathecal AD-h-IL10 was given over the lumbosacral spinal cord leading to elevated lumbosacral cerebrospinal fluid (CSF) levels of human IL-10 (around 11 ng/ml CSF). AD-h-IL10 attenuated both ipsilateral thermal hyperalgesia and bilateral allodynia induced by CCI between day 13 and 24 after CCI [116].

AAV is important for the application of AAV-based vectors for gene therapy [3,186,187]. Recombinant AAV supports a genomic/gene carrying capacity of roughly 6 kb, and the only viral elements remaining in the recombinant virus are the inverted terminal repeats in the distal ends of the genome, structures required for helper mediated replication and capsid packaging [188]. An adeno-associated viral (serotype II; AAV2) vector was created that encodes IL-10 gene. Intrathecal administration of an AAV2 vector encoding beta-galactosidase revealed that AAV2 preferentially infected meningeal cells surround the CSF space. Upon intrathecal administration, this AAV2-IL-10 vector was successful in transiently preventing and reversing chronic sciatic inflammatory neuropathy-induced mechanical allodynia for about 10 days [189].

LV belongs to a subclass of retroviruses that integrate into the host cell genome. Early LV vectors, based largely on HIV-1, include components of the HIV genome, but most of these elements have been removed in the newest generations [188]. LV vector-mediated gene delivery is one of promising methods for exploring pain pathophysiology and for genetic treatment of chronic neuropathic pain. Zou and colleague [190] reported that LV vectors delivering human IL-10 (LV/hIL-10) was administered intrathecally in rat CCI model of neuropathic pain. They found that intrathecal LV/hIL-10 reversed the enhanced pain states. GFP expression in spinal dorsal horn neurons 7 days after transduction with intrathecal delivery of LV/hIL10 was found. The concentration of hIL10 increased significantly after 3 days (1.68 ± 0.39 ng/mL) and 7 days (6.15 ± 2.66 ng/mL) of administration of LV/hIL10 but not after administration of LV/control. Intrathecal injection of LV/hIL10 significantly reversed CCI-induced mechanical allodynia and thermal hyperalgesia. IL-10 inhibits activation of the inflammatory HMGB1-RAGE pathway in the rat CCI model.

We constructed a non-replicating HSV-based gene transfer vector to express rat IL-10 for examining the effect of IL-10 expression in activated microglial cells *in vitro*, as well as in inflammatory pain *in vivo* [13]. Transfection of dissociated primary DRG neurons *in vitro* with vector QHIL10 resulted in robust expression and release of IL-10. Subcutaneous inoculation in the plantar surface of the hind paw of rats with 30 μl of QHIL10, resulted in expression of IL-10 in lumbar L4-L5 DRG and transport of vector-derived IL-10 protein to the central terminals of the pseudounipolar DRG axon in the dorsal horn of the lumbar spinal cord compared to the control vector that does not induce expression of IL10. IL-10 mediated by the HSV vectors reduced around 30% of the paw flinching (painful behavior) numbers in the formalin test [13]. The effect of IL-10 on nociceptive behavior correlated with a block in phosphorylation of p38 and reduced expression of TNFα in spinal microglia [13]. In a spinal cord injury (SCI)-induced pain model, at 1 week after injury animals with SCI demonstrated significant increases in pain-related behaviors including: mechanical hyperalgesia (withdrawal threshold 90.0 ± 5.86 g for sham vs. 52.3 ± 7.51 g for injured), thermal hyperalgesia (withdrawal latency 13.0 ± 1.42 sec for sham vs. 7.6 ± 0.28 sec for injured) and mechanical allodynia (threshold 11.7 ± 2.78 g in sham vs. 1.9 ± 0.28 g in injured) [191]. Subcutaneous inoculation of HSV vectors expressing IL10 one week after SCI reduced mechanical hyperalgesia (withdrawal threshold from 61.1 ± 3.75 g to 92.2 ± 5.44 g); thermal hyperalgesia (withdrawal latency from 7.58 ± 0.58 s to 12.5 ± 2.39 s); and mechanical allodynia (paw withdrawal threshold from 3.86 ± 0.71 g to 11.0 ± 3.85 g) by 2 weeks after injury, indicting the antinociceptive effect of the vectors expressing IL-10 [191].

Evidence shows that painful HIV sensory neuropathy is influenced by neuroinflammatory events that include the proinflammatory molecules, MAP Kinase, TNFα, stromal cell-derived factor 1-α (SDF1α), and CXC chemokine receptor type 4 (CXCR4) [192]. In the HIV glycoprotein gp120-induced neuropathic pain model, the hindpaws of rats were inoculated with the same vectors expressing IL-10 or the control vectors. The vectors expressing IL-10 resulted in a significant elevation of the mechanical threshold, and the area under curves (AUC) in QHIL10 was almost three times higher than that in the control vectors [193]. HSV vectors expressing IL-10 reversed the upregulation of phosphorylated p38 mitogen-activated kinase, TNFα, SDF1α, and CXCR4 expression at 14 and/or 28 days in the DRG and/or the spinal cord dorsal horn [193]. Similarly, in neuropathic pain induced by gp120 combined with 2′,3′-dideoxycytidine (ddC, one of the nucleoside reverse transcriptase inhibitors (NRTIs)), the hindpaws of rats were inoculated with the same vectors expressing IL-10 [194]. HSV vectors expressing IL-10 resulted in a significant elevation of mechanical threshold, and AUC in QHIL10 was around two times higher than that in the control vectors; the HSV vectors expressing IL-10 also concomitantly reversed the upregulation of p-p38, TNFα, SDF1α, and CXCR4 induced by gp120 with ddC in the lumbar spinal cord dorsal horn and/or the DRG [194].

#### P4.3.3. TNFSR

Glia activity and proinflammatory cytokine release (e.g., TNFα) are involved in spinal cord injury (SCI) [195]. TNFα plays an important role in the different neuropathic pain states [133]. TNFSR can prevent TNFα from the binding membrane TNF receptor, therefore, TNFSR reduces bioactivity of TNFα. In neuropathic pain induced by partial SCI, one week after subcutaneous inoculation of HSV vectors expressing TNFSR (T0TNFSR) into the plantar surface of the hind paws, TNFSR could be detected in neurons of the L4-L6 DRG ipsilateral to the inoculation site using immunocytochemistry and Western blot. The ipsilateral dorsal horn of the spinal cord showed substantial amounts of TNFSR in animals with HSV vectors expressing TNFSR, but not in animals with control vectors. The expression of TNFSR by HSV-mediated gene transfer reduced pain behavior and decreased number of ED1-positive cells, p-p38, and TNFα in the spinal cord dorsal horn [122]. Inoculation with either HSV TOTNFSR or control vector subcutaneously in the hind feet of uninjured rats, resulted in no change in either the mechanical or thermal threshold over the course of 3 weeks, indicating that expression of TNFSR does not alter basic sensory function in normal rats. Subcutaneous inoculation of T0TNFSR into the plantar surface of the hind foot ipsilateral to the ligation 1 week after SNL, resulted in a significant reduction in mechanical allodynia and thermal hyperalgesia that was apparent 1 week after inoculation. TNFSR achieved by the HSV vector-based gene transfer to the DRG, resulted in a reduction of TNFα, IL-1β, and phosphorylated p38 MAP kinase [121].

The application of HIV gp120 to the sciatic nerve induces mechanical allodynia and upregulates TNFα, CXCR4, and SDF1α in both the DRG and lumbar spinal cord dorsal horn [125]. HSV vectors producing TNFSR reversed mechanical allodynia, and suppressed the upregulation of TNFα, CXCR4, and SDF1α induced by gp120 in the DRG and spinal cord dorsal horn [125]. In painful diabetic neuropathy, proinflammatory cytokines are involved in this process. HSV vectors expressing TNFSR were inoculated subcutaneously into the footpad; western blot analysis of the DRG showed increased expression of TNFSR in rats with the T0TNFSR vectors compared to that with the control vectors at 7 days and 4 weeks confirming *in vivo* transgene expression of TNFSR by the vector [196]. The thermal pain thresholds of animals inoculated with the HSV TNFSR group was substantially improved (12.9 ± 1.2 s) compared to the diabetic alone or diabetic animals with control vectors (8.82 ± 0.4 s). T0TNFSR decreased TNFα and p-p38 in the spinal cord dorsal horn and DRG [196].

In the HIV-associated sensory neuropathy, neuropathic pain associated with the use of NRTIs in patients with HIV/acquired immunodeficiency syndrome is clinically common. We found that systemic ddC induced upregulation of TNFα, SDF1α, and CXCR4 in both the lumbar spinal cord and the L4/5 DRG [197]. Subcutaneous inoculation with T0TNFSR resulted in a statistically significant increase of the mechanical threshold that was apparent on day 3 after inoculation compared with the control vectors. The AUC in T0TNFSR was a 1.75-fold higher than that in the control vectors. T0TNFSR significantly upregulated the expression of TNFSR in the pooled L4/5 DRG and the spinal dorsal horn. TNFSR mediated by HSV vector reversed upregulation of TNFα, SDF1α, and CXCR4 induced by ddC in the lumbar spinal cord dorsal horn and the DRG [197].

#### P4.3.4. TNF shRNA

DRG-targeted gene delivery is a promising therapeutic option for the treatment of neuropathic pain. Ogawa and colleague [90] engineered a gene therapy strategy to relieve neuropathic pain by silencing TNFα expression in the dorsal root ganglion (DRG) using lentiviral vectors expressing TNF short hairpin RNA3 (LV-TNF-shRNA3) in mice. A mouse neuropathic pain model was induced by L5 spinal nerve transection (SNT). Immediately after transection, 1 μl (1.8 × 10^5^ IFU/μl) of lentiviral vector was injected onto the proximal transected site of the left L5 spinal nerve. GFP mRNA expression in the L5 DRG was induced by LV-shRNA3 on day 3, 7, 14 after SNT. LV-TNF-RNA3 suppressed TNFα expression in ipsilateral L5 DRG after SNT. Injection of LV-TNF-shRNA3 onto the proximal transected site significantly suppressed the mRNA levels of ATF3, NPY and IL-6, and reduced mechanical allodynia and neuronal cell death of DRG neurons.

Taken together, these results from several different groups of investigators provide proof-of-principle evidence that viral vector-mediated delivery of anti-inflammatory cytokines can provide an analgesic effect and set the stage for a human trial to treat chronic pain.

### P4.4. Anti-inflammatory cytokines mediated by viral vectors for morphine tolerance/withdrawal

Opioids are the main drugs for the treatment of acute and cancer pain, however, long-term administration of opioids produces negative health consequences, such as analgesic tolerance, dependence, and addiction [198]. The mechanisms of the negative health consequences are complicated. Chronic morphine exposure results in a strong upregulation of the glial markers and proinflammatory cytokines [115,138,140,199-201], and induces spinal p38 activation [202]. Intrathecal treatment with p38 inhibitor or minocycline prevents the development of morphine tolerance [202]. Co-infusion of chronic morphine and amitriptyline (a tricyclic antidepressant) can prevent morphine tolerance; daily injections of anti-IL-10 antibody blocked the anti-inflammatory effect of amitriptyline in the morphine/amitriptyline co-infused rats, suggesting that the anti-inflammatory effect of amitriptyline is through up-regulation of IL-10 in morphine tolerance [203]. Repeated infusion of morphine across the 5 days of the experiment resulted in a reduction of analgesic efficacy of morphine, or tolerance, in the control animals [200]. Lumbosacral intrathecal injections of replication-defective AD vector encoding for human IL-10 (AD-IL10) site-specifically induces high levels of human IL-10 protein in lumbosacral CSF; treatment with AD-IL10 reduced the development of tolerance to morphine during these days [200].

Subcutaneous inoculation of HSV vectors expressing TNFSR into the plantar surface of the hindpaws for 1 week upregulated the expression of TNFSR in the spinal dorsal horn. The vectors expressing TNFSR, enhanced the antinociceptive effect of acute morphine in rats, delayed the development of chronic morphine tolerance in rats at day 5-7 after chronic morphine, and reduced spinal TNFα and IL-1β induced by repeated morphine [115].

**Figure 5:**
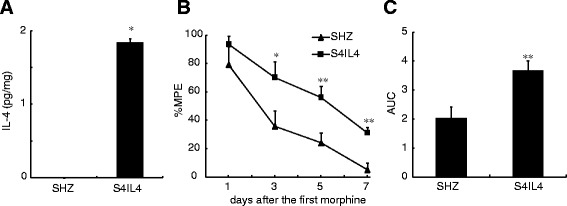
The expression of IL-4 mediated by HSV and its antinociceptive effect on morphine tolerance. **(A)** The expression of IL-4 mediated by HSV vectors S4IL4. Seven days after the vectors were inoculated into the hindpaws, the L4/5 DRGs were harvested. ELISA was conducted for testing the expression of IL-4 as described previously [12]. S4IL4 injection significantly induced the expression of IL-4. * *p* < 0.05 *vs* SHZ (control vectors), *t* test, n = 3-4. **(B)** The effect of S4IL4 and SHZ on chronic morphine tolerance. Seven days after the vector inoculation, rats received a repeated dose of morphine for 7 days. Thermal latency was tested at 60 min after each dose of morphine using a hotplate test. The percent maximum possible effect (MPE) of morphine in rats with S4IL4 was significantly higher than that with SHZ, * *P* < 0.05, **P <0.01 *vs.* SHZ, *t* test, n = 7. **(C)** The area under the curves in S4IL4 and SHZ were shown, **P <0.01 *vs.* SHZ, *t* test, n = 7.

**Figure 6:**
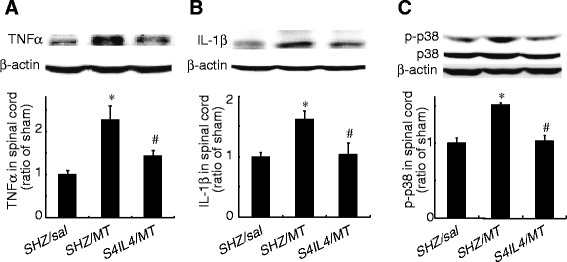
The effect of S4IL4 or SHZ on the expression of spinal TNFα, IL-1β, and p-p38. Rats were inoculated with S4IL4 or SHZ. Rats received a repeated morphine dose at 1 week after vector inoculation. In the sham group, rats received SHZ and a repeated dose of saline. At day 7, 1 hour after the last dose of morphine or saline, L4/5 spinal cord dorsal horns were harvested, and the expression of TNFα, IL-1β, and p-p38 were tested using Western blots. Repeated morphine administration significantly induced upregulation of spinal TNFα **(A)**, IL-1β **(B)**, and p-p38 **(C)**. The expression of spinal TNFα, IL-1β, and p-p38 in morphine tolerance rats with S4IL4, was significantly lower than that in morphine tolerance rats with SHZ. *P < 0.05 *vs* sham, # *P* < 0.05 *vs* SHZ/MT, n = 3-5, ANOVA, *post hoc* comparisons using Fisher’s PLSD test (StatViewJ 5.2).

Transgene-mediated expression of IL-4 mediated by the HSV vectors can suppress spinal c-Fos immunoreactivity, IL-1β, PGE2, and phosphorylated-p38 MAP kinase in neuropathic pain rats [12]. Since previous studies demonstrated that proinflammatory cytokines and/or p-p38 are involved in morphine tolerance [138,204], it is possible that IL-4 attenuates morphine tolerance in rats. The HSV vectors expressing IL-4 were injected into hindpaws of male Sprague-Dawley rats; 7 days after HSV vectors rats received chronic morphine (15 mg/kg, once a day, IP) for 1 week. Hot-plate thermal latency (52 ± 0.3°C) was performed with a Harvard Apparatus hot plate apparatus (Edenbridge, Kent, United Kingdom). We found the HSV-mediated IL-4 overexpression in the DRG using ELISA (Figure 5A), and IL-4 mediated by the HSV vectors significantly reduced antinociceptive tolerance to systemic morphine (Figure 5B). The area under the curves in S4IL4 was 3.68 ± 0.32 that was significantly higher than that in control vector SHZ (2.04 ± 0.36) (Figure 5C). We also observed that HSV-mediated IL-4 reversed the upregulation of TNFα, IL-1β, and p-p38 in the spinal cord dorsal horn in rats treated chronically with morphine (Figure 6). These results support the concept that proinflammatory molecules may play an important role in the pathogenesis of morphine tolerance induced by chronic morphine. These studies indicate that the ‘therapeutic’ use of non-replicative HSV-derived vectors containing immunomodulatory molecules might prove a novel approach to morphine tolerance.

Studies have implicated the midbrain periaqueductal gray (PAG) in the pathogenesis of morphine withdrawal, and recent evidence suggests that proinflammatory cytokines in the PAG may play an important role in morphine withdrawal [205]. We have reported that chronic morphine withdrawal-induced upregulation of glial fibrillary acidic protein (GFAP), TNFα and phosphorylation of ERK1/2 (pERK1/2) in the PAG [206]. HSV vector encoding TNFSR gene, ten days after a vector injection into the PAG significantly induced the expression of TNFSR in the PAG compared to non-expression of TNFSR by control vectors. HSV-based vectors expressing TNFSR microinjected into the PAG significantly reduced the naloxone-precipitated withdrawal behavioral response and downregulated the expression of GFAP and TNFα in astrocytes of the PAG. Microinjection of the HSV vectors expressing TNFSR into the PAG also reduced the phosphorylation of both ERK1/2 and CREB, and reduced Fos immunoreactivity in the PAG neurons following naloxone-precipitated withdrawal. These results support the concept that proinflammatory cytokines expressed in astrocytes in the PAG may play an important role in the pathogenesis of morphine withdrawal response [206].

### P4.5. Conclusions

Proinflammatory cytokines (e.g. TNFα, IL-1β) play an important role in neuropathic pain and chronic morphine tolerance/withdrawal. Non-replicative viral vectors expressing anti-inflammatory molecules (IL-4, IL-10 or TNF soluble receptor) might prove to be a novel approach to reducing neuropathic pain, morphine tolerance/withdrawal. Summary of characteristics of viral vectors expressing anti-inflammatory molecules for pain analgesia and morphine tolerance/dependence was shown in Table 1.

## Abbreviations

PHN: Post Herpetic Neuraglia
VZV: Varicella Zoster Virus
HSV: Herpes Simplex Virus
HPPE: Human preproenkephalin
TNF / TNFα: Tumor necrosis factor/tumor necrosis factor alpha
TRADD: TNF receptor associated death domain
TNFrsf21: Tumor necrosis factor receptor superfamily member 21
FDA: Food and Drug Administration
ZAP: Zoster Associated Pain
NSAID: Non-steroidal anti-inflammatory drug
IL-1: Interleukin 1
IL-6: Interleukin 6
ATF-3: Activating Transcription Factor 3
Na_v_: Voltage gated sodium channel
DNA: Deoxyribonucleic acid
MA: Mechanical Allodynia
TH: Thermal Hyperalgesia
NO: Nitric oxide
NMDA: N-methyl-D-aspartate receptor
ICP4: Infected cell polypeptide 4
ICP27: Infected cell polypeptide 27
GABA: Gamma-Aminobutyric acid
IL-1Β: Interleukin 1 beta
PGE2: Prostaglandin E2
MAPK: Mitogen-activated protein kinase
hCMV: Human Cytomegalovirus
pfu: Plaque Forming Units
DAMGO: [D-Ala^2^, N-MePhe^4^, Gly-ol]-enkephalin
NPY: Neuropeptide Y
RT-PCR: Reverse transcriptase quantitative polymerase chain reaction
SCIDhu: Severely Compromised Immunodeficient humanized
HIV: Human Immunodeficiency virus
gp120: Glycoprotein 120
dpi: Days Post Infection
